# Structure, reliability, and validity of the revised child anxiety and depression scale (RCADS) in a multi-ethnic urban sample of Dutch children

**DOI:** 10.1186/s12888-015-0509-7

**Published:** 2015-06-23

**Authors:** Mia P. Kösters, Mai J. M. Chinapaw, Marieke Zwaanswijk, Marcel F. van der Wal, Hans M. Koot

**Affiliations:** Department of Epidemiology & Health Promotion, Public Health Service of Amsterdam, P.O. Box 2200, 1000 CE Amsterdam, The Netherlands; Department of Public and Occupational Health, EMGO Institute for Health and Care Research, VU University Medical Centre, Van der Boechorststraat 7, 1081 BT Amsterdam, The Netherlands; NIVEL, Netherlands Institute for Health Services Research, P.O. Box 1568, 3500 BN Utrecht, The Netherlands; Department of Developmental Psychology, Faculty of Psychology and Education, VU University, Van der Boechorststraat 1, 1081 BT Amsterdam, The Netherlands; EMGO Institute for Health and Care Research, VU University Medical Centre, Van der Boechorststraat 7, 1081 BT Amsterdam, The Netherlands

**Keywords:** Anxiety, Depression, Youth self-report, Measurement properties, School children, RCADS

## Abstract

**Background:**

Although anxiety and, to a lesser extent, depression are highly prevalent in children, these problems are, difficult to identify. The Revised Anxiety and Depression Scale (RCADS) assesses self-reported symptoms of anxiety and depression in youth.

**Methods:**

The present study examined the factor structure, internal consistency, short-term stability, and validity including sensitivity to change of the RCADS in a multi-ethnic urban sample of 3636 Dutch children aged 8 to 13 years old.

**Results:**

Results indicate that the RCADS is a reliable and valid instrument. The original 6-factor structure was replicated to a fair extent in the present study (RMSEA = 0.048) and internal consistency was good (*α*s = 0.70-0.96). ICCs for short-term stability were 0.76 to 0.86. Girls and children who indicated wishing to participate in a program targeting anxiety and depression had higher RCADS scores. Sensitivity to change analyses showed that the RCADS can detect changes in anxiety and depression symptoms in children who participated in a preventive intervention. The study showed low agreement between teacher and self-reported internalizing problems, even for children scoring above the 90^th^ percentile of the RCADS, indicating a high level of problems, emphasizing the need to also take child reports into account when screening for anxiety and depression in children.

**Conclusion:**

This study shows that the RCADS can yield reliable data on a diversity of anxiety disorders and depression in urban children aged 8–13 from very diverse ethnic backgrounds.

**Trial Registration:**

Netherlands Trial Register: NTR2397. Registered 30 June 2010.

## Background

Anxiety disorders frequently occur in children, with lifetime prevalence of 15 to 20 % [1], and although depression is less prevalent in this age group (0.4 to 2.5 %), its prevalence rises quickly during adolescence [2]. Anxiety and depression are closely related. Children with an anxiety disorder are at increased risk for developing a depressive disorder [1, 3], while childhood-onset depressive disorder increases the risk for anxiety [3]. In addition to this, anxiety disorders in adolescence are associated with substance abuse/dependence and academic underachievement [1]. Moreover, childhood and adolescent depression are associated with poor outcomes in later life, including suicidal behavior, substance abuse, increased risk for other psychiatric disorders (e.g., bipolar disorder and personality disorders), and psychosocial, academic and work-related problems [3].

Despite the fact that anxiety and depression significantly hamper children’s current and future well-being, only a small percentage of children with these problems receive mental health care [4–6]. Children are dependent on parents or teachers if professional help is needed [6, 7]. Unfortunately, identifying children in need of treatment for anxiety or depression is difficult for others without information from the children themselves. Other informants may notice behavior that suggests emotional problems, such as crying or sad posture, but may be not well-informed on what children actually think or feel. This most likely explains the low correlations between children’s self-reported and teacher- or parent-reported internalizing problems found in an extensive meta-analysis [8]. Comparable results were reported in a Dutch general population sample of 10–11 year-old children [9]. When parent- and teacher-report of emotional problem behavior was examined in detail, it was found that parents and teachers did not recognize several of the symptoms reported by children. In practice, mental health professionals underline the usefulness of children’s report [10]. An instrument that can identify children in need of help, based on children’s own reporting, that can be used next to proxy-informants is therefore of utmost importance.

An instrument that may be useful for obtaining reliable information about anxiety and depression in youths is the Revised Child Anxiety and Depression Scale (RCADS). The RCADS is a self-report questionnaire with scales corresponding to the *Diagnostic and Statistical Manual of mental disorders* (DSM-IV, [11]) diagnostic criteria for anxiety and depressive disorders [12]. It has been shown to be a reliable and valid instrument in general population- and school-based samples in Australia, the Netherlands, Denmark and the United States [12–17], and in clinical- and school-based samples in Hawaii, USA [12, 14, 18]. The RCADS measures anxiety and depression symptoms separately, and is, in addition, the only self-report questionnaire for youth that measures symptoms of five different types of anxiety disorders. This is important because of the close relationship between these disorders. In contrast to other instruments, such as the Revised Children’s Manifest Anxiety Scale 2 [19] or the Children’s Depression Inventory 2 [20], the RCADS is freely available in various languages [21]. Due to budget constraints in both health care and research, free-of-charge questionnaires could enhance their use and therefore increase the possibility of identifying children with problems, as well as promoting research in the field of childhood anxiety and depression.

In this study we examined the factor structure, internal consistency, short-term stability, construct validity of the RCADS in school-aged children. Our study was conducted in a multi-ethnic urban sample of Dutch children, whereas samples in previous studies mainly consisted of Caucasian children from the more rural parts of the Netherlands [16, 22].

Construct validity, which refers to the extent to which the RCADS correlates with other similar constructs, was assessed in five ways [23]. Our first hypothesis was that the RCADS anxiety and depression scales would correlate positively, since anxiety and depression are closely related. A correlation around *r* = 0.7 was expected (e.g., [24]). The second hypothesis was that there would be moderate agreement between children scoring in the 90^th^ percentile of the RCADS scales and those scoring in the 90^th^ percentile of teacher-reported anxiety and depression. Children scoring in the 90^th^ percentile represent the most anxious or depressed children in the sample. Although research has shown that teachers may not identify every child with elevated anxiety and depression symptoms [8, 9], we expected that they are able to recognize the most anxious or depressed children. Mesman and Koot [9] found correlations of 0.30 for anxiety and depression between teacher and child report in a general population sample. As we only compared children with scores above the 90^th^ percentiles, we expected stronger agreement between child and teacher report. As a third aspect of construct validity, we investigated gender differences in RCADS scores. Female gender constitutes a risk factor for anxiety and depression. For depression however, gender differences start to occur in adolescence [1, 2]. Therefore, we expected that the girls in our sample would have significantly higher levels of anxiety symptoms, but because of the age of our sample (pre-adolescence), we expected smaller gender differences in depression than in anxiety. The fourth hypothesis was that children willing to participate in a prevention program addressing anxiety and depression would have significantly higher RCADS scores than children not willing to participate. Fifth, and finally, we examined sensitivity to change. An instrument for assessing emotional problems is regarded more useful if it not only indicates individual or group differences in symptom level or severity, but also is sensitive to changes in symptom levels (e.g. due to targeted interventions). Sensitivity to change has however not been examined in several other RCADS related papers. Mathyssek et al. [25] established in a general population adolescent sample longitudinal measurement invariance, which implies that changes in anxiety scores over time most likely reflect true changes. However, this study did not investigate the major depressive disorder (MDD) scale. Therefore, in this study we established sensitivity to change of the instrument using data from children participating in an indicated preventive intervention program, including the MDD scale.

Further, we investigated age differences in RCADS scores. As anxiety and depression increase in adolescence, we expected more symptoms of anxiety and depression in older children. These hypotheses are tested in the largest Dutch sample to date.

## Methods

### Participants and procedures

The present study is part of a controlled trial in which the effectiveness of an indicated school-based prevention program for childhood anxiety and depression is being evaluated [26].

All 265 primary schools in the Amsterdam area, the Netherlands, were asked to participate in the trial, 45 (17 %) of which expressed willingness to participate. The main reasons for declining participation were time constraints, other priorities or participation in other studies. Children and parents in participating schools received an information letter with a passive informed consent form. If parents or children did not wish to participate, they were free to withdraw. Children and teachers completed questionnaires (see Measures) in the classroom during school time. Researchers or research assistants explained how to complete the questionnaires and were available for additional clarification during completion of the questionnaire. Three months later, the RCADS was completed again by a subsample of children (children in control schools, see [26]) to assess its short-term stability. In addition, RCADS data were obtained from the intervention sample at baseline (T1), immediately after the intervention 10 weeks later (T2), and at 6- (T3) and 12-month (T4) follow-up. Children with elevated RCADS scores at T1 in intervention schools were invited to participate in an intervention targeting anxiety and depression. In control schools, parents of children with elevated anxiety and depression symptoms were notified after the trial (see [26]). The Medical Ethics Committee of the VU University Medical Center in Amsterdam, the Netherlands, approved the study protocol.

In total, 3890 children from grades 4, 5 and 6 of 45 primary schools were invited to participate in the trial. Parents of 3775 children consented to participate. No questionnaires were available for 139 children because they had left school, were ill during data collection or due to unknown reasons. The remaining sample of 3636 children (93 %) consisted of 1733 boys and 1898 girls (5 unknown), aged 8–13 years (*M* = 10.6, *SD* = 0.9) (21 unknown). Age was divided into four categories: 8/9 (10 %, *n* = 360), 10 (35 %, *n* = 1267), 11 (40 %, *n* = 1447) and 12/13 (15 %, *n* = 541). Ethnicity was based on the mother’s country of birth, or, if the mother was born in the Netherlands, the father’s country of birth [27]. Children were from diverse ethnic backgrounds: Dutch (40 %, *n* = 1438), Turkish (8 %, *n* = 302), Moroccan (15 %, *n* = 550), Surinamese and Antillean (11 %, *n* = 395), other Western (10 %, *n* = 354), other non-Western (13 %, *n* = 472), or unknown (3 %, *n* = 125). The Western group consisted of children from Europe (excluding Turkey), North America, Oceania, Japan and Indonesia (including former Dutch East Indies). The non-Western group consisted of children from Africa, Latin America and Asia (without Japan and Indonesia) [27]. These percentages are comparable to the distribution of ethnic groups among Amsterdam primary school children (37 %, 8 %, 18 %, 11 %, 11 %, 15 %, and 2 % in the 2011/2012 school year) [28]. The short-term stability subsample consisted of 1019 children (54 % girls), and the subsample in which self-report as well as teacher questionnaires were available consisted of 841 children (55 % girls).

### Measures

Children’s self-reported symptoms of anxiety and depression were assessed by the Revised Child Anxiety and Depression Scale (RCADS) [12]. The RCADS assesses symptoms of anxiety and depression through child self-report. It consists of 47 items, corresponding to childhood and adolescent anxiety and depressive disorders as defined by the DSM-IV. Items are summed into six subscale scores, i.e. generalized anxiety disorder (GAD), social phobia (SP), separation anxiety disorder (SAD), panic disorder (PD), obsessive compulsive disorder (OCD), and major depressive disorder (MDD). A total anxiety score consisting of all GAD, SP, SAD, PD, and OCD symptom scores can also be computed. Examples of items are: *“I worry when I think I have done poorly at something”*, *“I have trouble sleeping”* and *“I feel scared if I have to sleep on my own”*. Children rate how often each item applies to them on a 4-point Likert scale, ranging from 0 (never) to 3 (always).

Socio-demographic information was assessed by means of a questionnaire. Children were asked to fill out their own date and country of birth, and the country of birth of their parents. Children were also asked to indicate whether they wanted to participate in a prevention program addressing anxiety and depression.

Teacher reports of anxiety and depression symptoms were assessed by means of the Problem Behavior at School Interview (PBSI) [29]. The PBSI is a 42-item instrument that measures internalizing and externalizing problems in children as perceived by teachers. In the present study only the 12-item internalizing scale, consisting of a 5-item anxiety scale and a 7-item depression scale, was presented as a questionnaire, as has been done in previous research [30]. Teachers rated children on a 5-point Likert scale, ranging from 0 (never) to 4 (often). Examples of items are: *“This child is nervous or tense”*, *“This child has a lack of energy”* and *“This child is unhappy or depressed”*. In previous research, Cronbach’s alphas ranged from 0.79 to 0.81 for the anxiety scale and from 0.78 to 0.83 for the depression scale [31]. In the present study, Cronbach’s alphas were 0.90 for the overall PBSI internalizing scale, 0.83 for the anxiety scale, and 0.86 for the depression scale.

### Analyses

When one item was missing on a particular RCADS subscale, the item median of the total sample was imputed. Medians were chosen, as data were skewed to the right. Per RCADS subscale, approximately 100 of the 3636 children had one missing item.

To test whether the six subscales as defined by Chorpita et al. [11] could be replicated in our sample, a confirmatory factor analysis was performed using Amos version 19 (James L. Arbuckle, 2010). A maximum likelihood estimation procedure was used. Model fit was evaluated by means of two indices. The Root Mean Square Error of Approximation (RMSEA) represents the average difference between correlations observed among variables and those expected on the basis of model assumptions. It also takes into account model parsimony. A value < 0.05 is considered to indicate a close fit and a value ≤ 0.08 indicates an acceptable fit (see [32]). The Tucker-Lewis coefficient (TLI; Tucker & Lewis, 1973) indicates the overall fit of the proposed model relative to a null model, adjusting for model complexity. A value > 0.90 indicates an acceptable fit and a value > 0.95 indicates a close fit of the model [33].

All other analyses were carried out with SPSS Statistics version 19 (IBM SPSS Statistics, 2010). Cronbach’s alphas were used to assess internal consistency of the RCADS. Alphas were computed for the total group as well as for ethnic subgroups. Short-term stability was assessed by calculating intraclass correlation coefficients (ICCs) in a subsample of 1019 children who did not receive the intervention (children in control schools, see [26]). To test the sensitivity to change of the RCADS, change in scores between baseline and three follow-ups were tested in 339 children who participated in an intervention study of an indicated prevention program targeting anxiety and depression (see [26]), by means of paired sample *t*-tests, followed by calculation of Cohen’s *d*.

Since all RCADS scales were skewed to the right, gender differences are reported in medians and interquartile ranges. However, to enable comparisons with previous studies, we also report means and standard deviations. Correlations were calculated as Spearman’s rho. Agreement between 90^th^ percentiles was calculated as kappa. Differences between scores for help-seeking, wanting to participate, gender and age were analyzed with linear regression analyses with the anxiety or depression scores as dependent variable. Linear regression analyses were chosen since they show the exact differences in scale scores between groups. The data allowed us to conduct these analyses, as residual plots revealed no large deviation from normally distributed residuals.

## Results

### Confirmatory factor analysis

Confirmatory factor analysis (in which no cross-loadings of items across factors, nor correlated errors were allowed) showed that the six RCADS scales as established by Chorpita et al. [12] were replicable to a fair extent in the present dataset. An RMSEA of 0.048 was obtained, indicating a good fit. The other fit index was somewhat lower (TLI: 0.86), indicating a fit close to acceptable values. Factor loadings for the GAD subscale ranged from 0.49 to 0.84, for SP from 0.55 to 0.74, for SAD from 0.51 to 0.61, for PD from 0.52 to 0.69, for OCD from 0.45 to 0.72, and for MDD from 0.38 to 0.69.

### Reliability and short-term stability

Cronbach’s alphas of the RCADS scales ranged from 0.75 to 0.95 in the total sample, and from 0.70 to 0.96 in the ethnic subgroups, indicating good internal consistency (Table 1). The short-term stability was good with ICCs ranging from 0.79 to 0.86 (Table 1). For each RCADS scale, the scores were slightly lower at the 3 months retest.

**Table 1:** Internal consistency (Cronbach’s α) and 3 months stability (ICC) of RCADS scales in a multi-ethnic sample of Dutch children aged 8-13 years in the total sample and in each of the ethnic subgroups

Scale	Total sample	Dutch	Turkish	Moroccan	Surinamese/Antillean	Other Western	Other non-Western	ICC
	*n* = 3601^a^	*n* = 1424^a^	*n* = 299^a^	*n* = 547^a^	*n* = 392^a^	*n* = 352^a^	*n* = 469^a^	*n* = 1019^a^
RCADS	0.95	0.95	0.94	0.96	0.94	0.96	0.95	0.86
Anxiety	0.94	0.94	0.93	0.95	0.94	0.95	0.94	0.86
GAD	0.84	0.83	0.82	0.86	0.83	0.86	0.82	0.79
SP	0.86	0.85	0.84	0.88	0.84	0.88	0.85	0.84
SAD	0.75	0.76	0.72	0.78	0.72	0.78	0.70	0.81
PD	0.83	0.82	0.82	0.86	0.82	0.85	0.83	0.79
OCD	0.76	0.77	0.71	0.77	0.71	0.78	0.73	0.80
MDD	0.79	0.79	0.79	0.80	0.72	0.81	0.78	0.82

RCADS = Revised Child Anxiety and Depression Scale. GAD = Generalized Anxiety Disorder. SP = Social Phobia. SAD = Separation Anxiety Disorder. PD = Panic Disorder. OCD = Obsessive Compulsive Disorder. MDD = Major Depressive Disorder. Other Western = Europe (excluding Turkey), North America, Oceania, Japan, Indonesia (including former Dutch East Indies). Other non-Western = Africa, Latin America, Asia (without Japan and Indonesia). ^a^N may vary because of missing values per scale

### Validity

Our first hypothesis, a high correlation between the RCADS overall anxiety scale and the MDD scale, was confirmed, with an *r* = 0.69 for boys and *r* = 0.74 for girls.

The second hypothesis, moderate agreement between children scoring in the 90^th^ percentile on the RCADS scales and children scoring above the 90^th^ percentile on the PBSI scales, was not confirmed. The kappa agreement between the RCADS anxiety 90^th^ percentile and the PBSI anxiety 90^th^ percentile was 0.08 for boys (38 boys above the RCADS anxiety 90^th^ percentile, 35 boys above the PBSI anxiety 90^th^ percentile, 6 of whom identified by both) and 0.12 for girls (46 girls above the RCADS anxiety 90^th^ percentile, 36 girls above the PBSI anxiety 90^th^ percentile, 8 of whom identified by both), indicating low agreement between child and teacher reports. The kappas of the other subscales varied from −0.01 to 0.17, also indicating low agreement (Table 2). The agreement was somewhat higher for depression with a kappa of 0.19 for boys (37 boys above the MDD 90^th^ percentile, 37 boys above the PBSI depression 90^th^ percentile, 10 of whom identified by both) and 0.25 for girls (45 girls above the MDD 90^th^ percentile, 43 girls above the PBSI depression 90^th^ percentile, 14 of whom identified by both). Other kappas between the RCADS anxiety scales and PBSI depression scale varied from 0.04 to 0.21, also indicating low agreement.

**Table 2:** Agreement (kappa) between deviant (>90^th^ percentile) scores on RCADS and PBSI scales in a multi-ethnic sample of Dutch children aged 8 – 13 years for boys and girls separately

Scale	PBSI Anxiety	PBSI Anxiety	PBSI Depression	PBSI Depression
	Boys	Girls	Boys	Girls
	*n* = 381	*n* = 460	*n* = 381	*n* = 460
Anxiety	0.08	0.12	0.10	0.09
Generalized anxiety disorder	0.09	0.12	0.09	0.15
Social phobia	0.13	0.03	0.21	0.04
Separation anxiety disorder	0.17	0.08	0.10	0.06
Panic disorder	0.06	0.17	0.14	0.14
Obsessive compulsive disorder	0.09	0.12	0.15	0.12
Major depressive disorder	−0.01	0.09	0.19	0.25

RCADS = Revised Child Anxiety and Depression Scale. PBSI = Problem Behavior at School Interview

Our third hypothesis, that girls would have higher RCADS scores than boys, was confirmed. Median scores were significantly higher for girls than for boys (effect sizes ranging from 0.16 for MDD to 0.46 for SP), except for OCD (Table 3).

**Table 3:** Medians (interquartile ranges) and means (standard deviations) of the RCADS scales in a multi-ethnic sample of Dutch children aged 8-13 years in the total sample and by gender

Scale (range of scores)	Total sample		Boys		Girls	
	*n* =3601^a^		*n* = 1712^a^		*n* = 1885^a^	
	Med (IQR)	M (SD)	Med (IQR)	M (SD)	Med (IQR)	M (SD)
RCADS (0–141)	24 (25)	28.4 (20.2)	21 (22)	25.0 (17.9)	26 (28) ^b^	31.6 (21.6)
Anxiety (0–111)	19 (21)	22.8 (16.9)	16 (17)	19.7 (14.9)	21 (23) ^b^	25.6 (18.1)
GAD (0–18)	3 (4)	4.3 (3.6)	3 (5)	3.9 (3.4)	4 (5) ^b^	4.7 (3.7)
SP (0–27)	7 (7)	7.7 (5.2)	6 (6)	6.5 (4.6)	8 (7) ^b^	8.9 (5.5)
SAD (0–21)	2 (4)	2.7 (3.2)	1 (3)	2.1 (2.7)	2 (4) ^b^	3.3 (3.5)
PD (0–27)	3 (5)	4.2 (4.3)	2 (4)	3.6 (3.7)	4 (6) ^b^	4.8 (4.6)
OCD (0–18)	3 (5)	3.9 (3.5)	3 (5)	3.7 (3.3)	3 (5)	4.0 (3.6)
MDD (0–30)	5 (6)	5.7 (4.1)	5 (6)	5.3 (4.0)	5 (5) ^b^	6.0 (4.3)

RCADS = Revised Child Anxiety and Depression Scale. GAD = Generalized Anxiety Disorder. SP = Social Phobia. SAD = Separation Anxiety Disorder. PD = Panic Disorder. OCD = Obsessive Compulsive Disorder. MDD = Major Depressive Disorder. Med = Median; IQR = interquartile range; M = mean; SD = standard deviation. ^a^N may vary because of missing values per scale. ^b^Difference from boys is significant at *p* < 0.001

The fourth hypothesis, i.e. that children who were willing to participate in a program targeting anxiety and depression would score significantly higher on the RCADS than children who did not wish to participate, was also confirmed by linear regression analyses: the differences were 15.27 (95 % CI = 13.99-16.54, *R*^*2*^ = 0.140) for the total RCADS scale, 13.03 (95 % CI = 11.97-14.10, *R*^*2*^ = 0.145) for the total anxiety scale, 2.21 (95 % CI = 1.98-2.44, *R*^*2*^ = 0.094) for GAD, 3.97 (95 % CI = 3.64-4.30, *R*^*2*^ = 0.140) for SP, 2.01 (95 % CI = 1.80-2.22, *R*^*2*^ = 0.097) for SAD, 2.78 (95 % CI = 2.51-3.06, *R*^*2*^ = 0.104) for PD, 2.07 (95 % CI = 1.85-2.30, *R*^*2*^ = 0.088) for OCD, and 2.26 (95 % CI = 1.97-2.51, *R*^*2*^ = 0.072) for MDD. Differences indicated small (*R*^*2*^ > 0.01) or medium (*R*^*2*^ > 0.09) effects.

Finally, paired *t*-tests on repeated assessments of RCADS scores in children participating in the intervention study showed that scale scores significantly differed between the consecutive assessments. The changes between T1 and T4 were the largest, with effect sizes ranging from a medium to a large (Table 4). The changes between two consecutive measurements decreased over time, ranging from small to medium between T1 and T2 to negligible between T3 and T4.

**Table 4:** Sensitivity to change: effect sizes (Cohen’s d) between T1, T2, T3, and T4 in a subsample of children who received a preventive intervention targeting anxiety and depression

Scale	T1-T2	T1-T3	T1-T4	T2-T3	T2-T4	T3-T4
	ES	ES	ES	ES	ES	ES
RCADS	0.55	0.89	1.05	0.36	0.50	0.14
Anxiety	0.55	0.89	1.04	0.38	0.52	0.14
GAD	0.39	0.72	0.77	0.36	0.45	0.15
SP	0.63	0.91	1.07	0.34	0.46	0.16
SAD	0.38	0.66	0.76	0.28	0.38	0.13
PD	0.44	0.78	0.91	0.35	0.46	0.11
OCD	0.50	0.79	0.88	0.34	0.49	0.14
MDD	0.44	0.69	0.83	0.27	0.36	0.11

RCADS = Revised Child Anxiety and Depression Scale. GAD = Generalized Anxiety Disorder. SP = Social Phobia. SAD = Separation Anxiety Disorder. PD = Panic Disorder. OCD = Obsessive Compulsive Disorder. MDD = Major Depressive Disorder

### Age differences

All RCADS subscale scores, including the overall RCADS and anxiety scales, were significantly lower for the 11-year-olds compared with the 8/9 year olds (Table 5). Our hypotheses regarding higher scores with increasing age could therefore not be confirmed.

**Table 5:** Medians (interquartile ranges) and means (standard deviations) of the RCADS scales in a multi-ethnic sample of Dutch children by age groups

Scale (range of scores)	8-9 years old		10 years old		11 years old		12-13 years old	
	*n* = 354^a^		*n* = 1253^a^		*n* = 1439^a^		*n* = 538^a^	
	Med (IQR)	M (SD)	Med (IQR)	M (SD)	Med (IQR)	M (SD)	Med (IQR)	M (SD)
RCADS (0–141)	26 (28)	31.5 (21.6)	26 (27)	31.0 (21.4)	22 (23) ^b,c^	26.3 (19.0)	22 (22.75) ^b,c^	26.2 (18.5)
Anxiety (0–111)	21 (24)	24.9 (18.1)	20 (23)	24.9 (18.1)	17 (20) ^b,c^	21.1 (15.8)	17 (19) ^b,c^	21.0 (15.6)
GAD (0–18)	4 (4)	4.6 (3.7)	4 (5)	4.8 (3.9)	3 (5) ^b,c^	4.0 (3.3)	3 (5) ^b,c^	3.9 (3.2)
SP (0–27)	7 (7)	8.0 (5.3)	7 (7)	8.1 (5.4)	7 (6) ^c^	7.4 (5.1)	7 (6) ^c^	7.6 (5.2)
SAD (0–21)	2 (3)	3.3 (3.6)	2 (4)	3.3 (3.5)	1 (3) ^b,c^	2.3 (2.8)	1 (3) ^b,c^	2.1 (2.8)
PD (0–27)	4 (6)	4.8 (4.6)	3 (6)	4.5 (4.5)	3 (5) ^b,c^	3.9 (4.1)	3 (4) ^b,c^	3.9 (4.1)
OCD (0–18)	3 (5)	4.1 (3.6)	3 (5)	4.3 (3.7)	3 (4) ^b,c^	3.5 (3.2)	3 (4) ^b,c^	3.5 (3.3)
MDD (0–30)	6 (6)	6.6 (4.4)	5 (5) ^b^	6.0 (4.3)	5 (5) ^b,c^	5.2 (4.0)	5 (5) ^b,c^	5.3 (3.9)

RCADS = Revised Child Anxiety and Depression Scale. GAD = Generalized Anxiety Disorder. SP = Social Phobia. SAD = Separation Anxiety Disorder. PD = Panic Disorder. OCD = Obsessive Compulsive Disorder. MDD = Major Depressive Disorder. Med = Median; IQR = interquartile range; M = mean; SD = standard deviation. ^a^N may vary because of missing values per scale. ^b^Difference from age category 8/9 is significant at *p* < 0.05. ^c^Difference from age category 10 is significant at *p* < 0.05

## Discussion

In the present study, the structure, reliability and validity of the RCADS were investigated in a large urban, multi-ethnic sample of Dutch school-aged children.

First, we investigated whether the original factor structure of the RCADS (Chorpita et al., 2000) could be replicated in our sample. The results were not univocal. In general, factor loadings were good and comparable to previous research [17]. However, whereas one fit index (i.e. the RMSEA) indicated a close fit, the TLI was slightly below the cut-off value of a good fit. The present study is not the first that found lower values for one or more of these fit indices [13, 16, 17]. The close relation between the RCADS subscales may make it difficult to establish a clear factor structure. Previous research has found high comorbidity rates between different types of anxiety (e.g., [34]). Further, Ferdinand et al. [22] found no distinction between GAD, SP, SAD and PD in a general population sample of pre-adolescent children. For depression, which has a low prevalence in the age category of our sample, the MDD scale may reflect anxiety symptoms rather than symptoms of depression. The strong correlation between the anxiety and MDD scale in the present study as well as in previous (e.g., [35]) research also points in this direction.

The internal consistency was good for all RCADS scales. Cronbach’s alphas were comparable with other samples [12, 13, 17], and were comparable between ethnic groups, indicating reliability in these groups. The ICCs indicated good stability over three months and were fairly comparable to the ICCs in the study of Muris et al. [16] over a four-week period.

The RCADS scores of children who were willing to participate in a prevention program for anxiety and depression were significantly higher than the scores of children who did not want to participate in such a program. This may indicate that the RCADS is capable of identifying children who feel the need to participate in such a program.

In contrast to our expectations, we found low agreement between child- and teacher-reported 90^th^ percentile scores. Apparently, even children with very high self-reported anxiety or depression scores are not easily identified by their teacher. As other measures in the present study provide confirmation for the validity of the RCADS (CFA, reliability, remaining validity measures including sensitivity to change), these results seem to indicate relative insensitivity of teacher reports rather than low validity of the RCADS child reports (cf. [9]). An important message from our findings as well as from previous studies is that, when screening for childhood anxiety and depression in the school context, child reports are essential to include next to reports from teachers [10].

Gender differences between RCADS subscales were as expected. Girls reported more anxiety and depression symptoms than boys. Again, these findings are indicative of the validity of the RCADS.

Sensitivity to change analyses showed that the RCADS detects change in anxiety and depression symptoms in children who participated in a preventive intervention up to 12 months after participation. The biggest changes were detected between the pre-intervention measurement and the last one 1.3 years later. Changes between consecutive measurements became smaller over time. These results are in line with the expected course of symptom decrease after participation in an intervention. Therefore, the RCADS is suitable for screening purposes as well as for evaluating change.

In general, scores of anxiety and depression were lower with increasing age. Three studies report lower RCADS scores with increasing age in childhood as well [16, 17, 36]. The latter two studies – which included a broader age range – reported a decrease of symptoms until middle or late adolescence, after which mean scores increased. The age category of our sample – childhood/early adolescence – was probably too young to detect a decrease in symptoms.

Most studies on the RCADS reported means and standard deviations. However, in our study the RCADS scores were not normally distributed but skewed to the right, with most children scoring low, as was to be expected in a general population sample. Our results show that, by using means, levels of anxiety and depression in children are being overestimated. Therefore, medians are the preferred descriptives. To enhance comparison between studies, we also reported the mean values.

### Strengths and limitations

The present study is the first to investigate the structure, reliability, stability and validity of the RCADS in a large and ethnically diverse sample of children from the general Dutch population. Although internal consistency of the RCADS subscales was comparable between the various ethnic groups, the applicability of the RCADS in different ethnic groups should be studied in more detail. For instance, multi-group confirmatory factor analyses can be used to investigate whether RCADS items are answered comparably by various ethnic groups (measurement invariance; [17]). However, the extensive report of measurement invariance is beyond the scope of the present study.

One of our methods to investigate the validity of the RCADS was to compare teacher and child reports of the presence of symptoms of childhood anxiety and depression. Ideally, the validity of the RCADS should also have been studied by comparing the RCADS scores with another child report instrument. However, because of time constraints at schools, we could not administer more questionnaires to the children.

## Conclusions

This study confirmed that the RCADS is a reliable and valid questionnaire for a multi-ethnic late childhood population. The RCADS is suitable for screening as well as detecting change in symptoms over time. Our findings confirmed the importance of using children’s self-reports when screening for symptoms of anxiety and depression, as these problems tend not to be adequately identified by teachers.
